# The Surface Structure and Thermal Properties of Novel Polymer Composite Films Based on Partially Phosphorylated Poly(vinyl alcohol) with Aluminum Phosphate

**DOI:** 10.1155/2014/439839

**Published:** 2014-11-20

**Authors:** Asmalina Mohamed Saat, Mohd Rafie Johan

**Affiliations:** ^1^Universiti Kuala Lumpur, Malaysian Institute of Marine Engineering Technology, 32200 Lumut, Perak, Malaysia; ^2^Nanomaterials Engineering Research Group, Advanced Materials Research Laboratory, Department of Mechanical, Faculty Engineering, Universiti Malaya, 50603 Kuala Lumpur, Malaysia

## Abstract

Partially phosphorylated polyvinyl alcohol (PPVA) with aluminum phosphate (ALPO_4_) composites was synthesized by solution casting technique to produce (PPVA)_100−*y*_ − (ALPO_4_)_*y*_ (*y* = 0, 1, and 2). The surface structure and thermal properties of the films were characterized using Fourier transform infrared (FTIR) spectroscopy and thermogravimetric analysis (TGA). The results showed that the films have higher thermal stability with strong bonding between PPVA and ALPO_4_.

## 1. Introduction

The development of new polymer-inorganic composite materials has garnered much interest over the years due to their unique microstructures and physical and chemical properties, which are markedly different from other materials. Polyvinyl alcohol (PVA) is one of the most important polymeric materials in the industry as it is environmentally friendly and of low cost. PVA is a hydrophilic polymer which is frequently used as a matrix for a variety of inorganic particles. PVA provides a convenient route to prepare composites whereby the inorganic particles are dispersed to a high degree of uniformity and fineness. The addition of polyacids to water-soluble PVA produces hydrogen bonded complexes. In the case of strong phosphoric acids (PA), the reaction of PVA may produce partial reactions to cyclic phosphate esters [1]. The remaining replaceable hydrogen of the cyclic phosphate groups is ionized in water and the esterified polymer behaves as a polyelectrolyte. Furthermore, there is physical rubbery after esterification [2]. The phosphorylation of PVA with phosphoric acid (PA) in producing partially phosphorylated poly(vinyl alcohol) (PPVA) has attracted considerable interest in the applications such as fire-retardant materials [3–8], electrolyte [2, 9–12], membranes [13–15], metal chelating [16–18], paper making [19], sensors [20–23], synthetic bones/teeth [17, 24], and nanoparticle/nanocomposite [25–28].

PPVA increases the amorphous structure of the polymer by decreasing its glass transition temperature (*T*_*g*_) and melting temperature (*T*_*m*_) [29]. PPVA complexes possess favourable properties such as good film forming, ion exchange, conductivity, chemical resistance, and flammability. Aluminum phosphate (ALPO_4_) is used industrially as a high temperature dehydrating agent. In addition, ALPO_4_ also serves as a fluxing agent, binder, and catalyst in organic synthesis. ALPO_4_ exhibits a rich structural diversity in both dense and crystalline microporous series framework.

In this paper, the synthesis of PPVA-ALPO_4_ composite films is described and their surface structures are examined. The thermal decomposition behaviour of PPVA-ALPO_4_ composites films is also investigated and compared with the decomposition characteristics of pure PVA.

## 2. Materials and Methodology

PVA and PA/ortho-PA (85%) were supplied by R&M Chemicals while ALPO_4_ was obtained from Sigma-Aldrich. The first step in producing the polymer composites involved modifying the PVA surface. The fabrication system consisted of three-neck round-bottom flask, thermometer, heating mantel, magnetic stirrer, and reflux vessel. Following this, 6.6 g of PVA, deionized water, and PA was added into the three-neck round-bottom flask [28, 30]. The mixture was dissolved by heating at 90°C for 30 minutes and reflux was carried out by continuous stirring for 1 hour; ALPO_4_ was then added to produce the composites and the solution was maintained at 80–90°C under continuous stirring for 1 hour. The composition of various films is summarized in Table 1.

The complexation and chemical properties of all samples were analyzed using Perkin Elmer System 2000 Fourier transform infrared (FTIR) spectrometer with a scanning range of 4000–400 cm^−1^. The thermal properties of the samples were recorded using Mettler Toledo TGA/SDTA851 thermogravimetric analyzer with temperature range of 25–1000°C.

## 3. Results and Discussion

### 3.1. FTIR Analysis of PVA and PPVA Films

**Figure 1:**
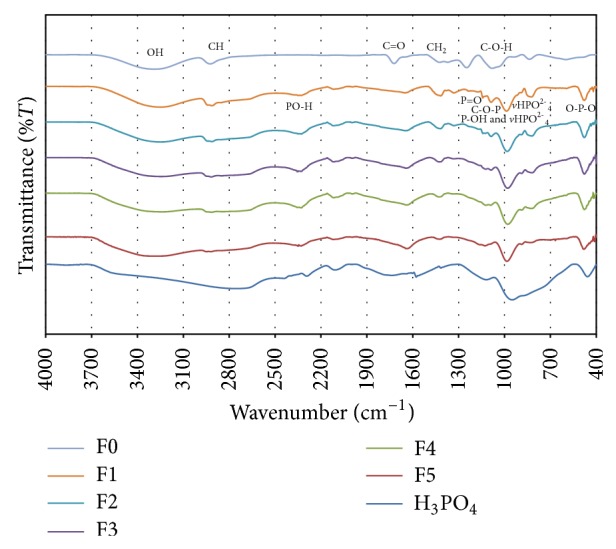
FTIR spectra of PVA and PPVA films.

The FTIR spectra for PVA/PPVA films (Samples F0–F5) are shown in Figure 1. The spectrum for pure PVA (F0) shows *ν*O–H (3288 cm^−1^), C–H (2925 cm^−1^), C=O (1722 cm^−1^), CH_2_ (1423 and 1247 cm^−1^), C–O–H (1080 cm^−1^) [7, 30], and skeleton (836 and 599 cm^−1^) [2] bands at the fingerprint region. The peak at 1722 cm^−1^ corresponds to the C=O stretching mode of the ester groups and occurs mostly in partially hydrolyzed PVA [31]. The stretching vibrations of carbonyl and/or carboxyl groups in the minor acetate groups (−CO(O)CH) in the PVA contribute to the existence of this peak [6]. In addition, there are intrainter molecular hydrogen bonds between the acetate groups in the PVA and adjacent OH group [32, 33].

It can be observed that the peaks for OH, P=O, and C–O–P bands disappear at higher concentrations of PA. Even though the intensity of the OH peak decreases abruptly upon the addition of PA, this intensity increases with an increase of PA due to the absorption of moisture by the film. The OH peak then broadens which indicates the interaction between PVA and PA [31] due to their phase separation and dehydration at higher acid concentrations. Variations in the P=O peak at 1329–1331 cm^−1^ for Samples F0–F2 are due to hydrogen bonding and the P=O peak disappears completely for Samples F3–F5. The C–OH peak disappears, whereas the C–O peak appears [7, 8, 30] upon the addition of PA. The disappearance of the C–OH peak is attributed to the chemical modifications of PVA by PA. The C–O–P peak shifts to higher wavenumber and attenuates due to hydrogen bonding. The overlapping of* v*P–O(C) and *v*HPO_4_^2−^ vibration groups [28, 30, 31] produces an intense peak. The C–O–P groups become weaker at higher concentrations of PA due to the formation of hydrogen bonds (*v*HPO_4_^2−^).

The peak intrinsic to C–H, PO–H, C=C, and C–O–P overlap with P–O, P–O, and O–P–O peaks is more apparent with an increase in PA concentrations. The C–H peak shifts to a higher wavenumber for Sample F3, whereas this peak shifts to a lower wavenumber for Sample F4. However, the peaks at 1634 and 1273 cm^−1^ which are not shifted correspond to the bending mode of water molecules [31] as well as PVA dehydration at higher concentration of PA [30]. The highest wavenumber recorded for the shift in O–H peak is 2355 cm^−1^ (Sample F3), whereas the lowest wavenumber recorded for this shift is 2338 cm^−1^ (Sample F4). It can be observed that there is increase in the intensity of peaks for the P–OH band [30] as well as in an overlapping of C–O–P and P–O, P–O, and O–P–O bands at higher concentrations of PA, which is attributed to the higher number of free PA molecules in the films. These peaks shift to higher wavenumbers. The peaks at 986–978 cm^−1^ belong to the P–O groups which originated from (H_2_PO_4_)^−^ and *v*HPO_4_^2−^ [28, 30, 31]. The emergence of the P–O peak can be observed at 825 cm^−1^ for Sample F2, whereas the *v*HPO_4_^2−^ peak shifts to a lower wavenumber as the PA concentration increases. However, the O–P–O peak at 477 cm^−1^ for the PPVA sample shifts to a higher wavenumber with an increase in PA concentration. The deformation of O–P–O as well as vibration modes in PO_4_^3−^ can be clearly observed [34].

**Figure 2:**
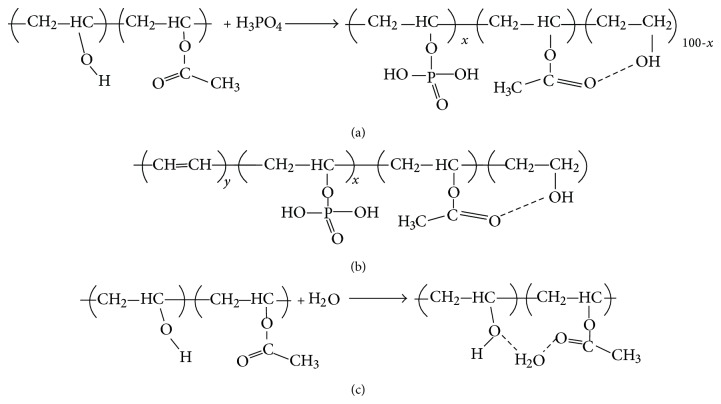


The maximum bonding number for PA to PVA occurs at a mole ratio of *R* = 0.33 (Figure 2(a)), which means that the three functional groups of PVA can react with one unit of PA. All protons are lost during the formation of C–O–P bonds at this value of *R*. However, it is expected that there will be free unreacted PVA groups for *R* values less than 0.33, due to insufficient free PA molecules. However, there will be additional free PA functional groups for *R* values above 0.33, giving none of the free PVA functional groups which will undergo a reaction. In this case, the free PA units will react with available OH groups, forming hydrogen bonds which will weaken and break up the C–O–P bond. An increase in HPO_4_^2−^ also weakens the C–O–P bonds. These bonds are stabilized by losing a proton H^+^ and forming a C=C bond which leads to dehydration, as observed in Sample F4. In this case, the C–O–P band shifts to a higher wavenumber (1122 cm^−1^) with an increase in intensity and a decrease peak width. However, the C=C band for Sample F4 shifts to a lower wavenumber with a decrease in intensity and an increase in peak width due to dehydration, while crosslinking of phosphorylation occurs in Samples F1–F3.

**Figure 3:**
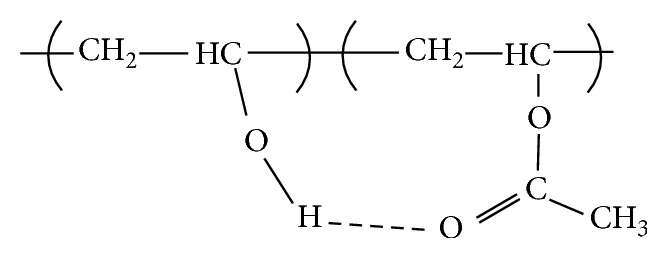
Intramolecular hydrogen bonding [33].

The nonlinear increase of PVA with PA molecules is attributed to the acetate groups in the PVA as well as plasticization effect of water on PVA [35]. However, the nonlinear increase tends to level off when *R* exceeds 0.33 or if the molar concentration of PA is 1.76 M [7]. Figure 2(a) shows the reaction between PVA and PA in order to produce partially phosphorylated PVA. The partially phosphorylated PVA consists of phosphorylated and unphosphorylated OH units as well as acetate groups with intermolecular bonding with adjacent OH. This agrees well with the FTIR results presented in Figure 3, in which partially phosphorylated PVA (Figure 2(a)) is produced for a mole ratio of *R* ≤ 0.33 whereas dehydration of PVA (Figure 2(b)) is produced for a mole ratio of *R* > 0.33 [30]. Dehydration of PVA produced conjugate double bond of the phosphonate groups in PVA [7]. The dehydration gel component increases, whereas the partially phosphorylated component decreases with increasing PA concentration due to the attenuation of C–O–P bond.

**Figure 4:**
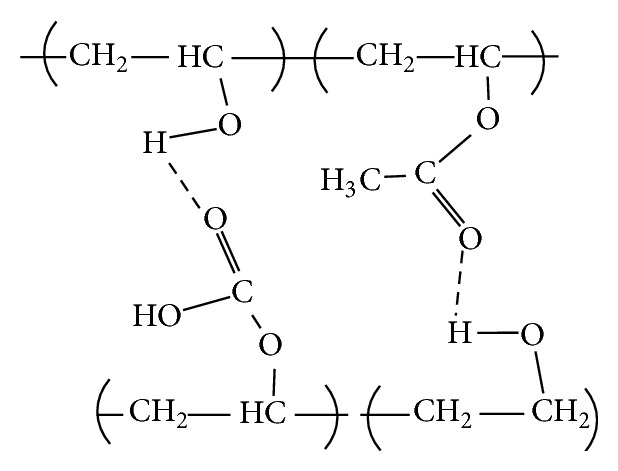
Intermolecular hydrogen bonding [33].

Figure 2(c) shows the C=O bond of partially hydrolyzed PVA, whereby bonding occurs with the OH groups in water [35]. The acetate groups only appear in pure PVA film (Sample F0), whereas the acetate groups have inter/intramolecular bond with available hydrogen in the PPVA films. The intramolecular and intermolecular hydrogen bonding is shown in Figures 3 and 4, respectively.

### 3.2. TGA Analysis of PVA and PPVA Films

**Figure 5:**
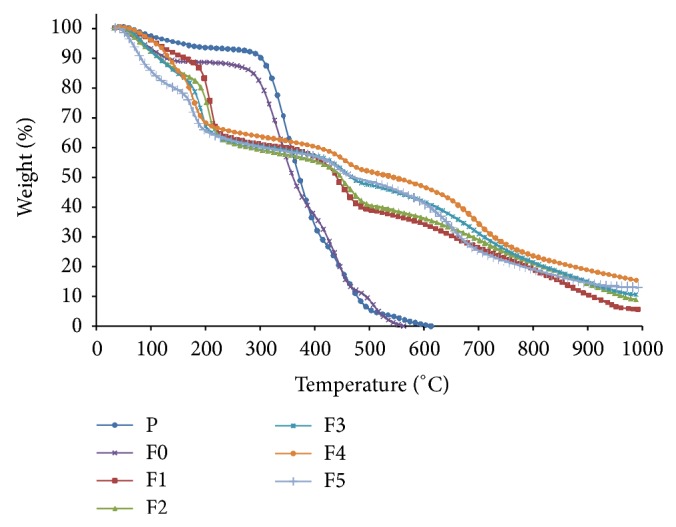
TGA curves of PVA powder (P), PVA (F0), and PPVA (F1–F5) films.

**Figure 6:**
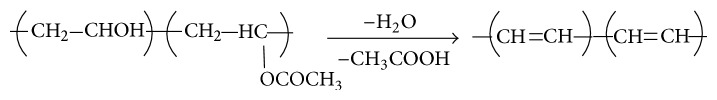


The TGA curves of PVA powder (P) and PVA-PPVA films (Samples F0–F5) are shown in Figure 5. The first weight loss is observed below 100°C, which is due to the evaporation of moisture. The first stage of degradation occurs between 130 and 270°C, whereby PVA begins to form a double bond as shown in Figure 6 as well as it start to decompose by elimination of water and acetate groups [35]. The partially hydrolyzed PVA releases acetic acids at a lower temperatures and decomposes at a higher temperature (330°C) [36]. The second stage of degradation occurs between 270 and 460°C due to the breakup of PVA backbones as well as degradation of acetate groups [35]. This stage is of particular interest in evaluating the thermal stability of the polymer. The third stage of degradation occurs between 460 and 600°C, whereby the PVA decomposes into impurities and other volatile materials. The PVA decomposes completely at 600°C.

The thermal degradation of PPVA produces a condensed phase mechanism which involves dehydration, crosslinking, and char formation [3]. The TGA curves for all PPVA samples are shown in Figure 5, and it can be observed that the PPVA samples have higher weight residue compared to PVA powder (P) and pure PVA film (F0). The degradation of complexed PPVA films begins at temperature below 100°C (first stage) due to the evaporation of water [37]. The second stage of degradation occurs between 120 and 190°C due to the elimination of water and volatiles products as well as formation of diphosphate [38] and triphosphate [7] and breakup of complexed PPVA [2]. The second stage of degradation occurs between 190 and 460°C due to the spontaneous degradation of PPVA and breakup of PVA backbones [35]. The decomposition of PVA begins at 460–700°C while the residue oxidation occurs between 700 and 950°C [8]. Char formation occurs during the final stage of degradation and the unoxidized residue remains at temperatures above 950°C [8].

**Figure 7:**
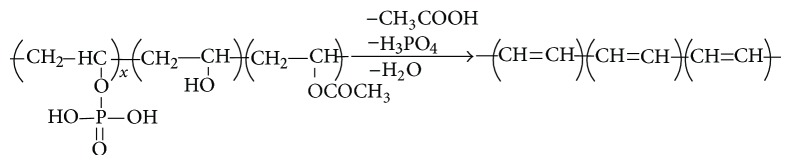


Spontaneous degradation of complexed PPVA films occurs at 190°C upon the addition of PA and the temperature continues to decreases to 170°C for the F5 film. Pyrolysis occurs at 168°C which is due to acid, whereas crosslinking between the phosphate groups and PVA occurs below 168°C [6]. The total degradation of complexed PPVA occurs at 950°C compared to pure PVA (600°C). Major degradation of PVA occurs during the second stage which constitutes 74% of the weight loss. In contrast, major degradation complexed PPVA occurs during the first stage with 32% weight loss. This clearly proves that Sample F3 attains the maximum bonding of PVA and PPVA. Below 90°C, PVA reacts with PA to form partially phosphorylated PVA known as polyvinyl alcohol phosphate (PVA-P) and tends to produce polyvinyl diphosphate (PVA-DP) at temperature above 90°C [38]. PVA-P is hydrophilic, whereas PVA-DP is hydrophobic. PVA-P can be produced using a reaction time less than 3 hours, whereas PVA-DP requires reaction times of more than 4 hours. PVA cannot be dissolved completely at temperatures less than 90°C. Consequently, the OH groups in PVA are not activated due to hydrogen bonding, which leads to esterification of PA to PVA at the surface of the PVA powder. PVA-P is mainly produced in Sample F3 in which a high weight loss can be observed due to water elimination and crosslinking of PVA-P to PVA-DP. The formation of PVA-P (hydrophilic) and PVA-DP (crosslink/hydrophobic) is influenced by the amount of water, reaction temperature, and reaction time. The phosphate groups that react with PVA in the autoclave yield favourable properties at reaction temperature 120°C compared to 70–90°C [26]. However, conjugated double bonds are formed when the PVA is heated above 120°C after the polymer experiences rapid chain-stripping elimination of water [25]. The weight residue increases in complexed PPVA. However the addition of PA reduces weight residue in the first stage of PPVA degradation. The degradation process of complexed PVA is given in Figure 7.

It can be seen that there is 14% weight residue due to water elimination and breakup of complexes for the PVA film upon the addition of 25 mL of water. Even though the amount of water decreases with an increase in PA concentration, the total solution (mixture of PA and deionized water) remains 25 mL which is the case for Sample F1. However, the weight residue for Sample F2 increases as the amount of water added decreases due to the formation of complexed PPVA which absorbs more moisture. Maximum bonding between PA and PVA occurs for Sample F3 with less water absorption. The maximum weight residue occurs during the first stage of degradation due to breakup of complexed PPVA. The PVA experiences dehydration from high concentrations of PA for Sample F4, whereby less water is absorbed. The plasticization effects of PA on PVA tends to level off and phase separation occurs at higher concentration of PA which is added into Sample F5 [39]. It is also observed that Sample F5 is sticky, wet, and oily.

### 3.3. FTIR Analysis of PPVA and PPVA-ALPO_4_ Films

**Figure 8:**
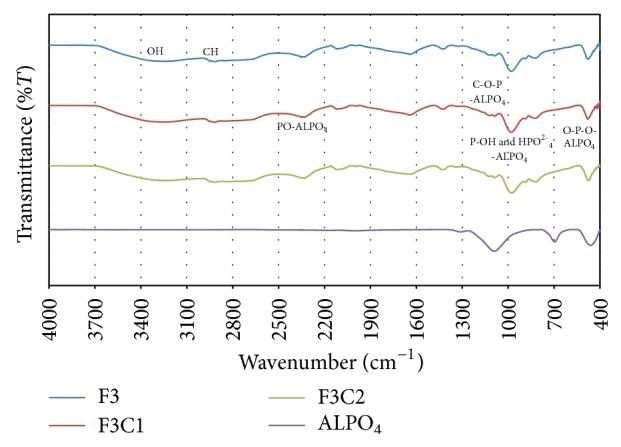
FTIR spectra of PPVA-ALPO_4_ composite films.

**Figure 9:**
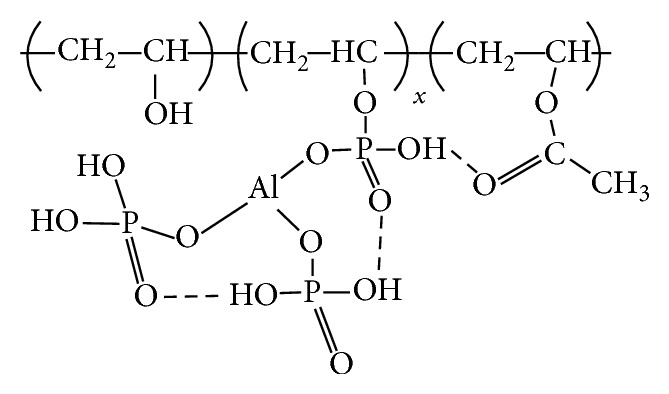
Model structure of PPVA-ALPO_4_ network.

The FTIR spectra for PPVA and PPVA-ALPO_4_ composite films are shown in Figure 8. The peak at 1088, 979, and 480 cm^−1^ for PPVA film (Sample F3) shifts to a lower wavenumber of 1086, 977, and 475 cm^−1^ for the PPVA-ALPO_4_ film (Sample F3C2) with a higher weight percent of ALPO_4_. The peaks are assigned to C–O–P, P–OH, *v*HPO_4_^2−^, and O–P–O bands, respectively, and indicate the interaction between PPVA and ALPO_4_. A similar trend was also observed for phosphate bonding interaction at C–O–P bands (1230, 1196, and 1166 cm^−1^), overlapping of C–O–P and P–O bands (930–970 cm^−1^), and O–P–O bands (603, 565 cm^−1^) with aluminum content [34]. The peak for the O–P–O band shows a decrease in intensity and broadening of peak width with an increase in ALPO_4_, which indicates a strong interaction between PPVA and ALPO_4_ by the formation of O–P–O–ALPO_4_ and C–O–P–ALPO_4_ bonds, as shown in Figure 9. The FTIR results are summarized in Table 2.

### 3.4. TGA Analysis of PPVA and PPVA-ALPO_4_ Films

**Figure 10:**
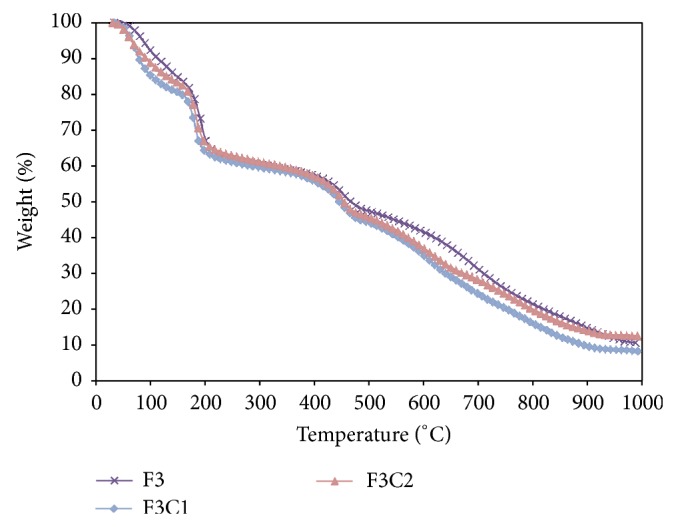
TGA curves for PPVA film (Sample F3) and PPVA-ALPO_4_ composite films (Samples F3C1 and F3C2).

The TGA curves for PPVA and composite PPVA-ALPO_4_ composite films are presented in Figure 10. It can be observed that the PPVA and PPVA-ALPO_4_ films have higher weight residue compared to the PPVA and PVA samples which is attributed to the higher crosslinking productions of diphosphate and triphosphate as well as reactions with ALPO_4_. This, in turn, increases char formation, which remains unoxidized in the waste. The thermal stability of the PPVA-ALPO_4_ composite film is improved compared to pure PVA and PPVA films due to the interfacial bonding between ALPO_4_ and PPVA. The pure PVA film is stable up to 260°C, whereas the PPVA and PPVA-ALPO_4_ samples are stable up to 190°C. Thermal decomposition occurs in two steps after water loss. Thermal decomposition begins at 220°C and ends at 492°C, which corresponds to the structure of the PVA. The first stage of degradation occurs at a faster rate, whereas the second stage of degradation is a slow process. The degradation of PPVA and PPVA-ALPO_4_ begins at a lower temperature compared to PVA. The amount of weight residue for PPVA-ALPO_4_ composite film is higher compared to PPVA and PVA films.

## 4. Conclusion

In this study, polymer-inorganic composites have been synthesized successfully by solution casting technique. The FTIR results reveal that the maximum bonding between PVA and PA occurs in the F3 film. The PPVA-ALPO_4_ composite film exhibits enhanced thermal properties compared to PPVA and PVA films. Weight loss begins at a lower temperature in PVA-ALPO_4_ and PPVA compared to pure PVA. The highest weight residue is obtained after thermal decomposition in air compared to PPVA and PVA. The thermal stability of PPVA and PPVA-ALPO_4_ films is significantly higher than that for pure PVA which proves a strong bonding between PPVA and aluminum phosphate.

## Figures and Tables

**Table 1:** Composition of PVA, PPVA and PPVA-ALPO_4_ films.

Sample code	PVA (M)	PA (M)	PA/PVA (R)	ALPO_4_ (%wt)
F0	0.15	—	—	—
F1	0.15	0.0172	0.1150	—
F2	0.15	0.0345	0.2299	—
F3	0.15	0.0517	0.3449	—
F4	0.15	0.0690	0.4599	—
F5	0.15	0.0862	0.5748	—
F3C1	0.15	0.0517	0.3449	1
F3C2	0.15	0.0517	0.3449	2

**Table 2:** Interpretation of PVA/PPVA and PPVA-ALPO_4_ FTIR spectra.

Band assignment and wavenumber (cm^−1^)														
Sample	OH	C–H	PO–H	PO–H	C=C	C=O	CH_2_	P=O	C–O–P	C–O–H	C–O–P, P–O	P–O	Sk	O–P–O
F0	3288	2925				1722	1423, 1247			1080			836, 599	
F1	3243	2913	2327	2116	1645		1417	1329	1087		986	825		477
F2	3252	2913	2328	2117	1645		1417	1331	1086		981	819		477
F3	3242	2917	2355	2117	1645		1422		1088		979	821		478
F4	3242	2915	2338	2116	1634		1424		1122		978	823		478
Intensity (%)	I	D	D	D	D		I	I	I		D	D		D
Peak position	L	L	H	U	U		H	H	H		L	L		U
**Peak size**	**S**	**S**	**B**	**B**	**B**		**S**	**S**	**S**		**B**	**B**		**B**
F3C1	3243	2918	2353	2117	1645		1422		1088		979	821		480
F3C2	3242	2913	2328	2117	1645		1429		1085		977	820		478
Intensity	I	U	U	U	D		U		D		D	D		D
Peak position	L	L	L	U	U		H		L		L	L		L
**Peak size**	**S**	**U**	**U**	**U**	**B**		**U**		**B**		**B**	**B**		**B**

Sk = skeletal, intensity, I = increase, D = decrease, peak position, L = shift to lower wavelength, H = shift to higher wavelength, U = unshifted, peak size, B = increase, S = decrease, U = unchanged.
